# A WeChat applet-based national remote emergency system for malignant hyperthermia in China: a usability study

**DOI:** 10.1186/s12911-023-02275-4

**Published:** 2023-09-05

**Authors:** Hong Yu, Lingcan Tan, Tao Zhu, Xiaoqian Deng

**Affiliations:** Department of Anesthesiology, West China Hospital, Sichuan University, Chengdu, 610041 China

**Keywords:** Malignant hyperthermia, Anesthesiology, mHealth, Usability testing, Survey methods, Reliability and validity

## Abstract

**Background:**

Malignant hyperthermia (MH) is a rare anesthetic emergency with a high mortality rate in China. We developed a WeChat applet–based National Remote Emergency System for Malignant Hyperthermia (MH-NRES) to provide a real-time emergency system to help Chinese anesthesiologists deal with MH crises. However, it is imperative that close attention should be paid to the usability of the applet.

**Purpose:**

The objectives of this study were to (1) evaluate the usability of the applet-based MH-NRES for anesthesiologists; and (2) to test the validity and reliability of a modified mHealth app usability questionnaire.

**Methods:**

A modified User Version of the Mobile Application Rating Scale (uMARS) was designed. Together with System Usability Scale (SUS) and Post-Study System Usability Questionnaire (PSSUQ), another two well-validated questionnaires, uMARS were then used to evaluate the usability of MH-NRES. The Cronbach alpha of the total score and the subscales of uMARS was calculated to evaluate the internal consistency. The correlation coefficients among three questionnaires were calculated.

**Results:**

In this study, 118 anesthesiologists provided responses to the questionnaire. The overall mean uMARS score was 4.43 ± 0.61, which ranged from 3 to 5. The mean PSSUQ score were in good to excellent range with mean of 6.02 ± 0.97, which ranged from 3.19 to 7. The overall SUS score was 76.0 ± 17.6, which ranged from 45 to 100. The total uMARS score had excellent internal consistency (Cronbach alpha = 0.984). uMARS and its subscales were strongly correlated with PSSUQ (coefficient 0.758–0.819, *P* < 0.001) and SUS (coefficient 0.535–0.561, *P* < 0.001), respectively.

**Conclusions:**

Data obtained from the usability evaluation questionnaires in this study indicated a high quality of the MH-NRES on the ease of use, satisfaction and perceived usefulness, which suggest this system might be a useful tool for anesthesiologists’ education and management of MH crises. Future feedback from high-fidelity simulation and clinical scenarios are need for further usability evaluation of this system.

**Supplementary Information:**

The online version contains supplementary material available at 10.1186/s12911-023-02275-4.

## Background

### Introduction

Malignant hyperthermia (MH) is a rare anesthetic emergency with an estimated incidence between 1/5,000 and 1/250,000 general anesthetics [1, 2]. MH is normally triggered in susceptible individuals by volatile anesthetics or succinylcholine [3] and manifested by life-threatening pharmacogenetic muscle disorder with abnormal hypermetabolic reactions [4]. Although the reported incidence of MH cases was low, the predicted prevalence of MH-related genetic mutations has been reported between 1/2000 and 1/3000, and even may be as high as 1/400 [5–7]. As a result, MH should not be neglected with the high genetic prevalence and its fatal characteristic. Between 1985 and October 2020, a total of 136 MH events occurred in mainland China with a mortality rate as high as 55.9% [8]. Early recognition and appropriate management without any delay, especially the administration of the only disease-specific drug (i.e. dantrolene) is the key to successful rescue [9]. However, the knowledge about diagnosis and treatment of MH is still lacking among anesthesiologists [10]. In addition, dantrolene is only stocked in a few of Chinese hospitals even after its commercially available in China after 2020 [8]. In order to solve the above problems, we developed the WeChat applet–based National Remote Emergency System for Malignant Hyperthermia (MH-NRES) [11]. This real-time emergency system can assist anesthesiologists to make rapid diagnosis, initiate dantrolene mobilization from other hospitals in China, execute effective treatment, and provide subsequent gene diagnostic services and online simulation training.

Concerns about the accuracy, reliability and efficacy of mobile health (mHealth) apps are often raised [12]. Therefore, prior to the MH-NRES for providing evidence-based MH information and education, and consequently improving clinical outcomes, it is imperative that close attention should be paid to the usability of the applet. Therefore, further research needs to be done to ensure that MH-NRES are appropriately designed and targeted to the anesthesiologists’ needs before they are used as health interventions. The usability questionnaire is the most frequently used methods for evaluation of mobile app usability, as its simplicity in terms of data collection and analysis. In practice, some studies use the well-validated usability questionnaires designed for general software systems such as the System Usability Scale (SUS) [13] and Post-Study System Usability Questionnaire (PSSUQ) [14]. As they were frequently and extensively used, the validity can be guarantee and certain usability of several aspects can be reliably assessed [15]. However, for some aspects that are unique to mHealth apps, these questionnaires cannot provide the specific information that the designers expected. As a result, many other studies created their own usability questionnaires according to the general guidelines [15–17]. Leanne Hides and colleagues developed the user version of the Mobile App Rating Scale (uMARS) which derived from the former Mobile App Rating Scale (MARS) to classify and evaluate the quality of mHealth apps [18, 19]. Considering the target users and the function of our system, we developed a modified usability questionnaire according to uMARS in our previous study [11] to evaluate the user experience and perception of the system.

### Objectives

In this study, the objectives were to conduct a usability evaluation of the MH-NRES using the newly modified uMARS, and to validate the modified mHealth app usability questionnaire by comparing to two widely used usability questionnaires, PSSUQ and SUS.

## Methods

### Study design and study participants

After the development of a fully functional WeChat-based prototype, we recruited anesthesiologists from hospitals nationwide to take part in usability evaluation of this system. We released questionnaires in the homepage of MH-NRES through the online survey tool Sojump (Shanghai Information Co.) during a period of two weeks. The demographic characteristics of the anesthesiologists were collected. After general introduction of the MH-NRES, study participants were asked to finish four tasks using this system, and provide responses to the modified version of uMARS, PSSUQ and SUS. Only after the participants had completed the four tasks can the system setting allow opening the questionnaire.

### Tasks performed by anesthesiologists in the usability study

**Fig. 1:**
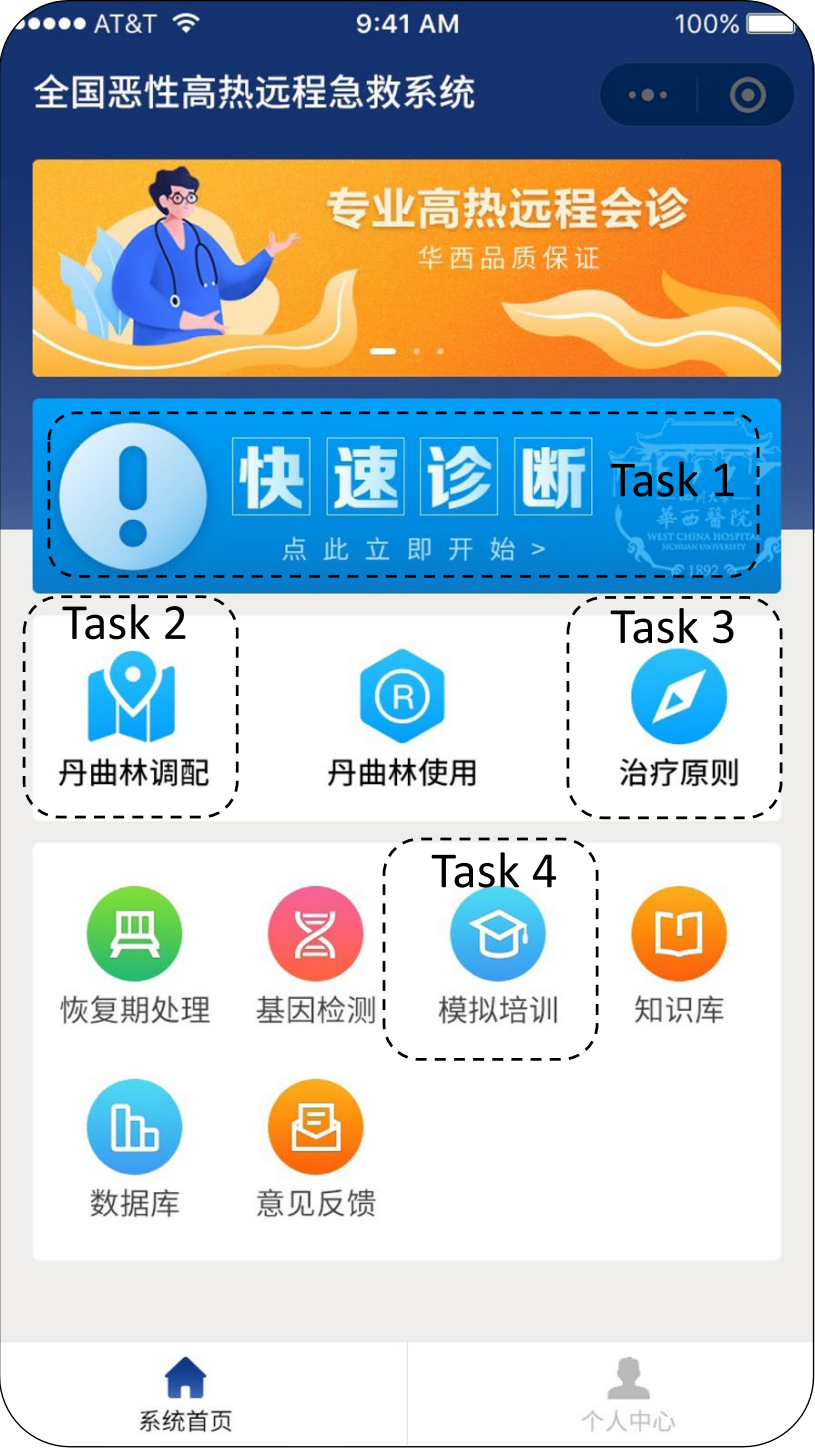
Tasks performed by anesthesiologists using the MH-NRES applet

When using the MH-NRES applet, participants were asked to finish the following tasks: (1) calculate the MH Clinical Grading Scale score of a simulative MH case in the Quick Diagnosis module of the system; (2) find a nearest drug suppliers in the Dantrolene Mobilization module; (3) learn the principles of treatment in the MH Treatment module; and (4) learn a MH case in the Online Simulation Training module (Fig. 1).

### Usability evaluation questionnaires

We simplified the modified version of uMARS mentioned in our previous study [11] and developed a new uMARS in this study. The new uMARS comprises 20 questions using a 5-point Likert scale with higher score corresponding to good usability. It consists of three domains: (1) system quality, including functionality (items 1–4), engagement (items 5–6), aesthetics (item 7), and content information (items 8–11) provided in the applet; (2) subjective quality: it questions likelihood of recommending the app to others (item 12), use in future (item 13) and overall rating (item 14); and (3) perceived impact: it assesses the perceived impacts on awareness (item 15), knowledge (item 16), confidence (item 17), and behavior change (items 18–20) (Additional file 1). We modified the new uMARS by excluding those questions that are not relevant to the current applet. For example, we omitted the items assessing entertainment and interest in the engagement domain because the applet was more possibly to be used as an aid when a potential clinical MH occurs. Also, we simplified the aesthetics domain by removing some questions that seemed repetitive and confusing. Besides, as a free and nonprofit property of the applet, we excluded the question “Would you pay for this applet?” in the subjective quality domain.

The PSSUQ used in this study comprises 16 items rated on a 1- to 7-point scale ranging from strongly disagree (1) to strongly agree (7). We defined items 2, 5, 7–10, 12 and 13 were the first subscale (PSSUQ1) which is related to system quality, items 1, 4, 14 and 16 were the second subscale (PSSUQ2) which is related to satisfaction, and items 3, 6, 11 and 15 were the third subscale (PSSUQ3) which is related to perceived impact (Additional file 2).

The SUS used in this study comprises a 10-item Likert scale for respondents with responses to the statements ranging from strongly disagree (1) to strongly agree (5) (Additional file 3) [20, 21]. Scores for odd items was converted by subtracting one from the user response, and for even-numbered items by subtracting the user responses from 5. After the conversion procedure, scores for each item were added together and then multiplied by 2.5 to create a single SUS score between 0 and 100. A SUS score above a 68 would be considered above average and supports acceptability for use [20].

### Statistical analyses

#### Descriptive statistics

A descriptive analysis was conducted on the demographic characteristics of the study participants. The means and standard deviation (SD) for individual statements of the entire scale and the three subscales of uMARS and PSSUQ were calculated, respectively. Then the mean and SD for the average value of the entire scale and the three subscales of uMARS and PSSUQ were then reported. Individual SUS scores were calculated for each study participant, and a mean SUS score with SD was provided for usability evaluation results.

#### Psychometric analysis

To evaluate the internal consistency of the total score and the subscales of uMARS, the values of Cronbach alpha was calculated for the entire questionnaire and its subscales. Cronbach alpha values of > 0.7 are acceptable [22]. To determine the criterion validity of the uMARS, the correlation coefficients among the scores of the uMARS, PSSUQ, and SUS and their subscales were calculated. Also, the inter-subscale correlation coefficient within the uMARS was to be calculated to determine the construct validity of the uMARS. Larger absolute values of correlation coefficients indicated a stronger relationship. The statistical significance of correlation coefficient was evaluated by correlation t-test. *P* values < 0.05 were considered statistically significant. All these statistical analyses were performed using SPSS statistical software (version 23.0; IBM Corp).

#### Ethics considerations

Institutional review board (IRB) approval was obtained from Ethics Committee in West China Hospital of Sichuan University (IRB registration number: 2021–1387). All the procedures of the study were followed in accordance with Declaration of Helsinki. Participants provided written informed consent prior to completing the survey.

## Results

### Participants

A total of 118 anesthesiologists completed the survey after 2 weeks of questionnaire release. All study participants provided responses to all the statements on the three usability questionnaires. The demographic characteristics of these participants are shown in Table 1.

**Table 1:** Demographic information of the study participants (*n* = 118)

Characteristic	Value
**Age (years), mean (SD)**	37.1 (8.1)
< 30	21 (17.8)
30–45	73 (61.9)
> 45	24 (20.3)
**Gender, n (%)**	
Male	61 (51.7)
Female	57 (48.3)
**Education, n (%)**	
Associate degree	9 (7.6)
Bachelor’s degree	25 (21.2)
Master’s degree	83 (70.3)
Doctoral degree	1 (0.9)
**City/Province, n (%)**	
Northwest China	76 (64.4)
Southwest China	16 (13.6)
North/Northeast China	9 (7.6)
East/South/Central China	17 (14.4)
**Hospital level, n (%)**	
Tertiary hospital	87 (73.7)
Non-tertiary hospital	31 (26.3)

### Usability evaluation

The overall mean uMARS score was 4.43 ± 0.61, which ranged from 3 to 5. The mean system quality score was 4.38 ± 0.62; the mean satisfaction score was 4.46 ± 0.66. The perceived impacts on awareness, knowledge, confidence, and behavior had a mean score of 4.51 ± 0.64 (Table 2). The PSSUQ score were in good to excellent range with mean of 6.02 ± 0.97, which ranged from 3.19 to 7. The mean score of subscales for system quality was 6.00 ± 0.99, for satisfaction 6.07 ± 0.99, and perceive impact 6.02 ± 1.01. The overall SUS score was 76.0 ± 17.6, which ranged from 45 to 100, which was above the acceptable score of 68, indicating high usability.

**Table 2:** Detailed results of the user version of the Mobile App Rating Scale

Subscale	Mean (SD)	Minimum	Maximum
Overall mean	4.43(0.61)	3	5
System quality	4.38(0.62)	3	5
Functionality	4.37(0.64)	2.75	5
Engagement	4.42(0.64)	3	5
Aesthetics	4.31(0.75)	2	5
Information	4.40(0.65)	3	5
Satisfaction	4.46(0.66)	3	5
Recommend this system to others	4.50(0.69)	3	5
Use this system in the future	4.42(0.74)	2	5
Overall star rating	4.46(0.69)	3	5
Perceived impact	4.51(0.65)	3	5
Awareness	4.50(0.66)	3	5
Knowledge	4.49(0.69)	3	5
Confidence	4.52(0.68)	3	5
Behavior change	4.51(0.66)	3	5

### Reliability and validity of the uMARS

The total uMARS score had excellent internal consistency (Cronbach alpha = 0.984). Internal consistencies of its subscales were also very high (objective quality alpha = 0.975; subjective quality alpha = 0.927; perceived impact alpha = 0.978). The correlation coefficients among the scores of the uMARS, PSSUQ, SUS and their subscales were shown in Table 3. The table showed that the three subscales in uMARS were correlated (coefficient 0.843–0.973, *P* < 0.001). In addition, uMARS and its subscales were strongly correlated with PSSUQ (coefficient 0.758–0.819, *P* < 0.001), PSSUQ1 (coefficient 0.784–0.815, *P* < 0.001), PSSUQ2 (coefficient 0.747–0.802, *P* < 0.001), PSSUQ3 (coefficient 0.723–0.784, *P* < 0.001) and SUS (coefficient 0.535–0.561, *P* < 0.001), respectively. These correlation coefficient values show the criterion validity and construct validity of the uMARS.

**Table 3:** Correlation coefficients among scores from the uMARS, PSSUQ, SUS and their subscales

Scales	uMARS1	uMARS2	uMARS3	uMARS	PSSUQ1	PSSUQ2	PSSUQ3	PSSUQ
uMARS2	0.875							
uMARS3	0.843	0.901						
uMARS	0.973	0.942	0.939					
PSSUQ1	0.808	0.754	0.748	0.815				
PSSUQ2	0.787	0.748	0.747	0.802	0.972			
PSSUQ3	0.784	0.723	0.727	0.770	0.940	0.954		
PSSUQ	0.808	0.760	0.758	0.819	0.993	0.987	0.967	
SUS	0.535	0.555	0.535	0.561	0.571	0.549	0.540	0.569

*PSSUQ* Post-Study System Usability Questionnaire, *SUS* System Usability Scale, *uMARS* user version of the Mobile App Rating Scale

## Discussion

The aim of this study was to evaluate the usability of the first real-time MH emergency system in China, the MH-NRES, with three questionnaires (uMARS, PSSUQ and SUS) and to validate a simpler version of uMARS including usability components of system quality, satisfaction and perceived impact. All responses of three questionnaires indicated good system usability. Also, uMARS and its subscales were strongly correlated with PSSUQ and SUS.

The evaluation tool used by the end users was of particular importance. In our study, we used questionnaire simplified and summarized from a user version of MARS, which was established for the general population to identify high quality mobile apps [11]. This new questionnaire was highly reliable which was reflected in the excellent internal consistency and correlation with PSSUQ and SUS, two well-validated usability questionnaires designed for general software systems. The feedback of uMARS indicated a high system quality including appropriate target population, user-friendly operability and high-quality information. System quality refers to the whole performance of an mHealth app as perceived by the users, which is the prerequisite for ensuring that users can easily to learn and use the system and obtain the information they need [23]. Low-quality information of a system may mislead users and influence users’ perception of its usefulness [24, 25]. In addition, the study participants provided feedback of high degree of satisfaction, and perceived usefulness on awareness, knowledge, confidence as well as behavior change related to MH management. User satisfaction refers to a user’s emotional state about using an mHealth app which is considered to be associated system quality [23]. Perceived usefulness refers to users’ beliefs about the effectiveness and benefits of using an mHealth app which predicts user satisfaction [26, 27]. These views may well explain the excellent internal consistency found among the system quality, satisfaction and perceived impact using the uMARS questionnaire in our study. A recent cross-sectional study found that perceived usefulness of the app was one of the most notable factors associated with smartphone medical app use by physicians [28]. It was reinforced by another study which found that perceived usefulness and user satisfaction contributed to patients’ intention to make continuous use of mobile health app [29]. Therefore, findings from the usability evaluation in the current study suggest that this system is both useful and welcomed by anesthesiologists.

Assessment scores obtained from all three different questionnaires in our study were comparable or higher than the scores for other health-related mobile apps [30–32]. The reason of the prominent positive feedback may be attributed to a number of reasons. MH is a low-frequency, high-risk situation, many anesthesiologists are unprepared to deal with the crisis due to rare clinical experience and merely relying on text knowledge in China [10]. To our knowledge, the WeChat applet–based MH-NRES is the first smartphone medical app to help anesthesiologists to deal with MH crises in China. The MH-NRES is a very practical applet with well-organized information and charactered by providing experts’ hotline, dantrolene mobilization, self-diagnosis and treatment instructions, which is completely different from other emergency system of MH (ie, the WeChat help groups) [11]. In addition, compared to MHAUS, a nonprofit organization who provides a 24-h MH hotline to give real-time advice in managing MH crises, this system is more suitable for Chinese users because of the problem of language barrier and absence of the approach to dantrolene mobilization. By reason of the foregoing, it makes sense that respondents give promising feedback to this system.

There were some strengths and limitations in the study. The current usability test provided strong evidence that the WeChat applet-based MH-NRES is a promising tool to help anesthesiologist to deal with MH crisis and consequently improving clinical outcome. Also, the study provided a validated version of uMARS with strong construct validity and criterion validity, which can be applied to evaluate similar mHealth app for other clinical emergencies. The limitation of the study was that the performance aspects of usability evaluation was lacking. The reason was that MH is a rare anesthetic emergency, therefore, the usability test did not assess the use experience among anesthesiologists in real clinical situations. However, this system is designed not only used in clinical crises scenarios, but also providing MH related knowledge to make anesthesiologists to be well-prepared before a real MH crisis occurs. The current study could be considered as a preliminary usability evaluation, and further feedback on the usefulness of this system from anesthesiologists who encounter MH crisis in high-fidelity simulation scenario or real operating room environment are needed.

## Conclusions

Data obtained from the usability evaluation questionnaires in this study indicated a high quality of the MH-NRES on the ease of use, satisfaction and perceived usefulness, which suggest this system might be a useful tool for anesthesiologists’ education and management of MH crises. Future feedback from high-fidelity simulation and clinical scenarios are need for further usability evaluation of this system.

### Supplementary Information

**Additional file 1.** Modified user-version of Mobile Application Rating Scale (uMARS).

**Additional file 2.** Post-Study System Usability Questionnaire (PSSUQ).

**Additional file 3.** The System Usability Scale (SUS).

## Data Availability

The datasets generated and analyzed during the current study are available from the corresponding author on reasonable request.

## Abbreviations

MH: Malignant hyperthermia
MH-NRES: National Remote Emergency System for Malignant Hyperthermia MHAUS: Malignant Hyperthermia Association of the United States
mHealth: Mobile health
PSSUQ: Post-Study System Usability Questionnaire
SUS: System Usability Scale
UI: User interface
uMARS: User version of the Mobile App Rating Scale
