# Supplementary material for: A WeChat applet-based national remote emergency system for malignant hyperthermia in China: a usability study
Source: BMC Med Inform Decis Mak. 2023 Sep 5;23:175. doi: 10.1186/s12911-023-02275-4 (PMC10478249; doi:10.1186/s12911-023-02275-4)
Supplement: Supplementary file 1 — Additional file 1. Modified user-version of Mobile Application Rating Scale (uMARS). [file 12911_2023_2275_MOESM1_ESM.pdf]

Additional file 1. Modified user-version of Mobile Application Rating Scale (uMARS)

System Quality Ratings (1=inadequate, 5 =excellent)

A. Functionality

Q1. Performance: How accurately/fast do the applet features (functions) and components (buttons/menus) work?

Q2. Ease of use: How easy is it to learn how to use the applet; how clear are the menu labels/icons and instructions?

Q3. Navigation: Is moving between screens logical/accurate/appropriate/ uninterrupted; are all necessary screen links present?

Q4. Gestural design: Are interactions (taps/swipes/pinches/scrolls) consistent and intuitive across all components/screens?

B. Engagement

Q5. Interactivity: Does it allow user input, provide feedback, contain prompts (reminders, sharing options, notifications, etc.)? Note: these functions need to be customizable and not overwhelming in order to be perfect.

Q6. Target group: Is the applet content (visual information, language, design) appropriate for your target audience?

C. Aesthetics

Q7. Visual appeal: How good does the applet look?

D. Information–Contains high quality information (e.g. text, feedback, measures, references) from a credible source

Q8. Quality of information: Is applet content correct, well written, and relevant to the goal/topic of the applet?

Q9. Quantity of information: Is the information within the applet comprehensive but concise?

Q10. Visual information: Is visual explanation of concepts-through charts/graphs/images/videos, etc. - clear, logical, correct?

Q11. Credibility of source: does the information within the applet seem to come from

a credible source?

Satisfaction Ratings

Q12. Would you recommend this applet to people who might benefit from it?

1 I would not recommend this applet to anyone

2 There are very few people I would recommend this applet to

3 There are several people I would recommend this applet to

4 There are many people I would recommend this applet to

5 I would recommend this applet to everyone

Q13. How likely do you think you would use this applet if it was relevant to you?

1 Not at all

2 Between 1 and 3

3 Maybe

4 Between 3 and 5

5 Definitely

Q14. What is your overall(star) rating of the applet?

1 ★

2 ★★

3 ★★★

4 ★★★★

5 ★★★★★

Perceived impact (1=strongly disagree, 5 =strongly agree)

Q15. Awareness–This applet has increased my awareness of the importance of MH

Q16. Knowledge – This applet has increased my knowledge/understanding of the MH

Q17. Confidence – The applet has increased my confidence to manage of MH crisis

Q18. Behavior change – This applet would encourage me to seek further help to improve the management of MH (if I needed it)

Q19. Behavior change – Use of this applet will help me to have access to obtain dantrolene in an MH crisis

Q20. Behavior change – Use of this applet will help me to improve the management of MH
