# Supplementary material for: A WeChat applet-based national remote emergency system for malignant hyperthermia in China: a usability study
Source: BMC Med Inform Decis Mak. 2023 Sep 5;23:175. doi: 10.1186/s12911-023-02275-4 (PMC10478249; doi:10.1186/s12911-023-02275-4)
Supplement: Supplementary file 2 — Additional file 2. Post-Study System Usability Questionnaire (PSSUQ). [file 12911_2023_2275_MOESM2_ESM.pdf]

Additional file 2. Post-Study System Usability Questionnaire (PSSUQ)

The PSSUQ is a 16-item questionnaire with 7 response options (1=strongly disagree, 7 =strongly agree).

1. Overall, I am satisfied with how easy it is to use this system.
2. It is simple to use this system.
3. I am able to complete my work quickly using this system.
4. I feel comfortable using this system.
5. It was easy to learn to use this system.
6. I believe I became productive quickly using this system.
7. The system gives error messages that clearly tell me how to fix problems.
8. Whenever I make a mistake using the system, I recover easily and quickly.
9. The information (such as on-line help, on-screen messages, and other documentation) provided with this system is clear.
10. It is easy to find the information I need.
11. The information is effective in helping me complete my work.
12. The organization of information on the system screens is clear.
13. The interface of this system is pleasant.
14. I like using the interface of this system.
15. This system has all the functions and capabilities I expect it to have.
16. Overall, I am satisfied with this system.
