# Supplementary material for: A WeChat applet-based national remote emergency system for malignant hyperthermia in China: a usability study
Source: BMC Med Inform Decis Mak. 2023 Sep 5;23:175. doi: 10.1186/s12911-023-02275-4 (PMC10478249; doi:10.1186/s12911-023-02275-4)
Supplement: Supplementary file 3 — Additional file 3. The System Usability Scale (SUS). [file 12911_2023_2275_MOESM3_ESM.pdf]

Additional file 3. The System Usability Scale (SUS)

The SUS is a 10-item questionnaire with 5 response options (1=strongly disagree, 5 =strongly agree).

1. I think that I would like to use this system frequently.
2. I found the system unnecessarily complex.
3. I thought the system was easy to use.
4. I think that I would need the support of a technical person to be able to use this system.
5. I found the various functions in this system were well integrated.
6. I thought there was too much inconsistency in this system.
7. I would imagine that most people would learn to use this system very quickly.
8. I found the system very cumbersome to use.
9. I felt very confident using the system.
10. I needed to learn a lot of things before I could get going with this system.
